# Foodborne urinary tract infections: a new paradigm for antimicrobial-resistant foodborne illness

**DOI:** 10.3389/fmicb.2013.00029

**Published:** 2013-03-06

**Authors:** Lora Nordstrom, Cindy M. Liu, Lance B. Price

**Affiliations:** ^1^Division of Pathogen Genomics, Center for Food Microbiology and Environmental Health, The Translational Genomics Research InstituteFlagstaff, AZ, USA; ^2^Department of Pathology, Johns Hopkins University School of MedicineBaltimore, MD, USA; ^3^School of Public Health and Health Services, George Washington UniversityWashington, DC, USA

**Keywords:** antibiotics, antibiotic resistance, antimicrobial resistance, *Escherichia coli*, food contamination, poultry products, UPEC, urinary tract infections

## Abstract

Urinary tract infections (UTIs) are among the most common bacterial infections worldwide. Disproportionately affecting women, UTIs exact a substantial public burden each year in terms of direct medical expenses, decreased quality of life, and lost productivity. Increasing antimicrobial resistance among strains of extraintestinal pathogenic *Escherichia coli* challenges successful treatment of UTIs. Community-acquired UTIs were long considered sporadic infections, typically caused by the patients’ native gastrointestinal microbiota; however, the recent recognition of UTI outbreaks with probable foodborne origins has shifted our understanding of UTI epidemiology. Along with this paradigm shift come new opportunities to disrupt the infection process and possibly quell increasing resistance, including the elimination of non-therapeutic antimicrobial use in food-animal production.

## INTRODUCTION

Public health concerns over the widespread non-therapeutic use of antimicrobials in food-animal production (FAP) have been voiced repeatedly since the 1960s, but little has been done to address these concerns in the US (FDA, 2012a). While some of the seminal research on the selection and transfer of antibiotic-resistant bacteria from food-animals to humans was conducted on *Escherichia coli* (Levy, 1978), the public health burden has largely been measured based on the classic foodborne pathogens, *Campylobacter* and *Salmonella*. However, today, we recognize that there is also frequent zoonotic transfer of antibiotic-resistant *Staphylococcus aureus* and *E. coli* from food animals to humans, and we must consider these events when attempting to quantify the full impact of antimicrobial use in FAP.

Traditionally, foodborne infections were limited to those affecting the gastrointestinal tract, but a growing number of studies linking foodborne *E. coli* with urinary tract infections (UTIs) challenge that narrow definition and have led us to adopt the term: foodborne UTI or FUTI |foō-tē|. In this mini-review, we discuss the predominant role and public health burden of *E. coli* in UTIs; the growing challenge of antimicrobial resistance to the successful treatment of UTIs; how the recognition of UTI outbreaks has spurred renewed interest in the role of foodborne *E. coli* in human disease; and the ramifications of the FUTI paradigm on antibiotic use policy in FAP.

## THE PUBLIC HEALTH BURDEN OF URINARY TRACT INFECTIONS

Urinary tract infections are the most common bacterial infections in the developed world, and among infections, only upper respiratory infections account for more hospitalizations each year (Mazzulli, 2002; Russo and Johnson, 2003). All ages are affected by UTIs and pediatric cases account for over a million office visits and 500,000 emergency-department visits per year (Spencer et al., 2010). Due to anatomical differences and the hormonal milieu of the urinary tract, women are significantly more likely than men to develop a UTI, and nearly half of all women will have a UTI during their lifetime (Foxman, 2003). When taking into account all age groups, the annual number of uncomplicated UTI cases is 6–8 million in the US and 130–175 million worldwide, and the vast majority of these cases are caused by *E. coli* (Russo and Johnson, 2003). The resultant costs associated with community-acquired UTI in the US approach $1.5 billion (Foxman, 2003).

Most cases of UTIs only involve the bladder (i.e., cystitis or lower UTIs) and resolve without sequelae; however, the infection can ascend to infect the kidney (i.e., pyelonephritis or upper UTI; Scholes et al., 2005). Although less frequent than cystitis, pyelonephritis affects approximately 250,000 individuals in the US each year (Russo and Johnson, 2003). Repeated cases of pyelonephritis, particularly in children, can cause scarring of the kidneys, leading to renal hypertension and even kidney failure later in life (Orellana et al., 2004).

Bloodstream infections originating in the urinary tract (urosepsis) are common and serious complications of UTIs. Urosepsis is more common in nosocomial UTIs (up to 12% of all nosocomial cases), but community-acquired urosepsis – particularly from antimicrobial-resistant *E. coli* – is occurring with increasing frequency (Rodriguez-Bano et al., 2006; Lee et al., 2012). The timely treatment of urosepsis is critical to reducing mortality, but the increasing prevalence of antibiotic-resistant *E. coli* limits clinical options and delays appropriate therapy (Rodriguez-Bano et al., 2010).

## ANTIMICROBIAL RESISTANCE IN *E. coli*, THE MAJOR UROPATHOGEN

*Escherichia coli* is the single most common uropathogen, causing 75–95% of all uncomplicated cystitis and pyelonephritis cases in the United States (Hooton, 2012). The *E. coli* strains that cause UTIs are known as extraintestinal pathogenic *E. coli* (ExPEC) and are genotypically and phenotypically distinct from non-pathogenic commensal *E. coli* and from diarrheagenic *E. coli*, which cause gastrointestinal infections (Johnson, 1991; Russo and Johnson, 2000). While ExPEC can colonize the human gastrointestinal tract similar to other *E. coli*, they are uniquely associated with infections outside of the gut, including: meningitis (Kim, 2012), osteomyelitis (Johnson and Russo, 2002; Lee et al., 2010), peritonitis (Bert et al., 2010), pneumonia (Johnson et al., 2003), sepsis, and, as discussed, UTI. In contrast to the diarrheagenic *E. coli* subgroups, where antimicrobials have limited utility, antimicrobials are critical for treating ExPEC infections.

As a general rule, antibiotics are considered no longer efficacious once the prevalence of resistance reaches 20% in a given population (Warren et al., 1999; Gupta et al., 2011). Thus, the general rise in antimicrobial-resistant and multidrug-resistant (MDR) *E. coli* worldwide has made clinical management of UTIs much more challenging (Smith et al., 2008; Johnson et al., 2009; Bahadin et al., 2011; Bosch et al., 2011; Okesola and Aroundegbe, 2011; Cullen et al., 2012). A survey of 1,729 human and food-animal *E. coli* isolates from 1950 to 2002 showed the overall MDR prevalence increased from 7.2% in the 1950s to 63.6% in the early 2000s (Tadesse et al., 2012). This increase was particularly marked among the food-animal isolates, which showed significant increases in resistance against 11 out of 15 antimicrobials tested, including ampicillin, trimethoprim-sulfamethoxazole (TMP-SMZ), cefoxitin, gentamicin, and amoxicillin/clavulanic acid (Tadesse et al., 2012).

The increasing challenge of antimicrobial resistance is epitomized by the global increase in *E. coli* that possess extended-spectrum beta-lactamases (ESBLs), a suite of enzymes that confer resistance to cephalosporins and monobactams (Woodford et al., 2004; Nicolas-Chanoine et al., 2008; Hoban et al., 2011; Johnson et al., 2012; Matsumura et al., 2012). In *E. coli*, ESBL genes are typically found on mobile genetic elements, which facilitate the carriage, accumulation, and transfer of antimicrobial-resistance genes. This is well illustrated by the ST131 clonal group – one of the most clinically important ExPEC lineages – that carries ESBL genes within transposon-like structures encoded on mobilizable plasmids (Peirano and Pitout, 2010; Canton et al., 2012; Matsumura et al., 2012). Additional antimicrobial resistance genes often co-localize on these plasmids, including elements conferring resistance to fluoroquinolones, aminoglycosides, and TMP-SMZ (Johnson et al., 2010; Pitout, 2012).

Another important mechanism facilitating the increase in antimicrobial-resistant UTIs is the introduction and clonal expansion of competitive, resistant *E. coli* strains in the community. These events appear to contribute more to the resistant UTI burden than *de novo* selection of resistant strains through clinical antimicrobial use (Smith et al., 2008). A longitudinal study of antimicrobial-resistant *E. coli* revealed that resistant populations were comprised of relatively few *E. coli* clonal groups and not the diverse population that would be expected from frequent *de novo* selection. In this study and others, the cessation of TMP-SMZ use did not decrease TMP-SMZ resistance; further suggesting that clinical antimicrobial use is not the sole driver of antimicrobial-resistant *E. coli* UTIs (Enne et al., 2001; Smith et al., 2008; Sundqvist et al., 2010).

Although the molecular and epidemiological mechanisms for the amplification of antimicrobial-resistant UTIs are complex, antimicrobial resistance in *E. coli* has largely followed antimicrobial use trends in human medicine and animal production, and with that, the clinical community has seen the loss of multiple antimicrobial classes against *E. coli* (Warren et al., 1999; Gupta et al., 2011). Tetracycline was introduced to clinical medicine in 1948 and served as a first-line therapy for UTIs until growing resistance gradually reduced its utility (Datta et al., 1971). Ampicillin was routinely used to treat UTIs after being introduced in 1961, but it also fell from favor due to exceedingly high resistance rates (NARMS, 2010). In 1999, the Infectious Disease Society of America (IDSA) declared ampicillin and amoxicillin unsuitable for treating UTI due to poor efficacy (Warren et al., 1999). Similarly, the emergence of fluoroquinolone-resistant *E. coli* is now limiting the utility of fluoroquinolones for treating patients with UTIs (Wang et al., 2001; Zervos et al., 2003; Christiansen et al., 2011; Kamenski et al., 2012; Longhi et al., 2012).

The increase in antimicrobial resistant UTIs in the community setting has also added caveats to standard clinical practice guidelines. The 2011 IDSA recommendation indicated that nitrofurantoin or TMP-SMZ should be used as first line treatments for uncomplicated UTI; however, the statement was issued with the qualification that TMP-SMZ should not be used if local resistance rates exceed 20% (Gupta et al., 2011). Indeed, many clinicians now rely almost exclusively on nitrofurantoin due to the growing TMP-SMZ resistance (Manges et al., 2001; Burman et al., 2003; France et al., 2005; Hooton, 2012; Vellinga et al., 2012).

## DISCOVERY OF UTI OUTBREAKS

Historically, UTIs were considered sporadic infections, but we now recognize that UTIs can also occur in outbreaks. In 2010, George and Manges (2010) conducted a systematic review of outbreak and non-outbreak studies involving *E. coli*. From 1950 to July of 2009, 12 *E. coli* UTI outbreaks were identified, with the first outbreak reported in 1986 and the latest in 2008. Nine of these 12 outbreaks occurred in Europe, including two in the UK (Phillips et al., 1988; Woodford et al., 2004), three in Spain (Prats et al., 2000; Oteo et al., 2006; Blanco et al., 2009), and one each in Denmark (Olesen et al., 1994), Portugal (Mendonca et al., 2007), and Croatia (Vranes et al., 2008). Three other outbreaks were reported in North America, with two in the US (Kunin et al., 1993; Manges et al., 2001) and one in Canada (Pitout et al., 2005). Although most outbreaks were limited to upper and lower UTIs, other infections were also reported, including urosepsis (Phillips et al., 1988; Woodford et al., 2004; Pitout et al., 2005; Oteo et al., 2006; Mendonca et al., 2007). These outbreaks were first recognized as temporal clusters based on susceptibility profiles, but the clonality of the *E. coli* isolates was not established until later using molecular analyses. Since most UTI *E. coli* isolates are not genotyped, the true incidence of UTI outbreaks is likely to be substantially under-reported (Stamm, 2001).

The definitive sources for these reported UTI outbreaks remain largely unknown. In some cases, outbreaks were postulated to have healthcare origins (Oteo et al., 2006; Mendonca et al., 2007; Woodford et al., 2007). In other cases imprudent antibiotic prescribing patterns in the community and lack of patient compliance were postulated (Oteo et al., 2006; Mendonca et al., 2007). However, the phenotypic and genotypic similarities of the causative *E. coli* strains is inconsistent with *de novo* selection, and, instead, suggest common point sources, such as contaminated food products (Olesen et al., 1994; Manges et al., 2001, 2008; Pitout et al., 2005).

## HUMAN COLONIZATION AND INFECTION WITH ANTIMICROBIAL-RESISTANT FOODBORNE *E. coli*

Antimicrobial-resistant *E. coli* from contaminated food can transiently colonize the human gastrointestinal tract and create a reservoir for subsequent infection (Bettelheim et al., 1977; Corpet, 1988; Johnson et al., 2007). One study showed a significant decrease in the concentration of tetracycline-resistant Enterobacteriaceae in participants’ stools after they switched from a normal diet to one consisting solely of sterilized food (Corpet, 1988). Another study, comparing human and poultry-associated *E. coli*, showed that the antimicrobial-resistant *E. coli* isolates from human subjects were genetically more similar to poultry isolates than to the susceptible *E. coli* strains coexisting in their own gastrointestinal tracts. These studies suggest that handling or ingestion of poultry was the primary source of resistant *E. coli* among the human subjects (Johnson et al., 2007).

In 2010, the National Antimicrobial Resistance Monitoring System (NARMS) reported that more than 75% of chicken and turkey, 59% of ground beef, and 40% of pork products tested in the US were contaminated with *E. coli*, and that a large portion of this foodborne *E. coli* was MDR (NARMS, 2012). While NARMS does not typically differentiate between pathogenic and non-pathogenic *E. coli* as part of their retail meat program, a recent study of *E. coli* from NARMS found that more than 20% of the isolates from chicken and turkey products, 8.3% from pork chops, and 3.4% from ground beef met the molecular criteria for ExPEC (Xia et al., 2011). Many of these ExPEC were MDR and belonged to the same phylogroups (B2 and D) as those most commonly associated with extraintestinal human disease (Xia et al., 2011).

Similar genetic fingerprints have also been found between temporally and geographically matched UTI cases and *E. coli* from food (Johnson et al., 2007; Vincent et al., 2010). Specifically, antimicrobial resistance and virulence gene profiles between phylogroup B2 *E. coli* from UTI cases were more closely related to *E. coli* from food animals and retail meat than from healthy human controls in Europe (Jakobsen et al., 2011). Another study from Canada showed similar findings (Bergeron et al., 2012). Additionally, clonal phylogroup D *E. coli*, which is frequently associated with UTI, was shown to be more common among poultry products than other types of meat (Vincent et al., 2010).

Other studies have shown that foodborne *E. coli* are not only genetically related to those causing UTIs in humans, they are also capable of causing UTIs *in vivo* (Jakobsen et al., 2010a,b). In a study conducted by Jakobsen et al. (2010b), all 13 foodborne phylogroup B2 *E. coli* strains tested in a murine UTI model led to lower UTIs in the animals and nine of the isolates also caused pyelonephritis. A similar investigation of 25 CgA isolates from poultry meat, chickens, and humans showed that all CgA strains from meat or food animals caused lower UTIs, and all but one of the isolates produced kidney infections (Jakobsen et al., 2010a).

To determine whether meat and poultry consumption is associated with development of antibiotic-resistant UTIs, a case–control study was initiated in which the dietary habits of women with MDR UTIs were compared with women with antimicrobial-susceptible UTIs (Manges et al., 2007). In this study, women with MDR UTIs were 3.7 times more likely to report frequent consumption of chicken, while those with ampicillin or cephalosporin-resistant infections were more 3.2 times more likely to report pork consumption (Manges et al., 2007).

## FOODBORNE URINARY TRACT INFECTIONS: A NEW PARADIGM

Taken together, the studies reviewed above provide compelling evidence that retail meat, particularly poultry, serves as an important reservoir for human exposure to antibiotic-resistant *E. coli* that is causing UTIs. Thus, the term foodborne UTIs or FUTIs has been adopted to describe these infections. The traditional mode of foodborne diseases necessarily involves an infection or toxification of the gastrointestinal tract; however, in FUTIs, the etiologic agent causes no gastrointestinal pathologies. Likewise, with classic foodborne infections, ingestion is the rate-limiting step: if a susceptible host consumes a sufficient dose of a pathogenic microbe, disease will ensue. The FUTI model requires at least two steps: (1) a susceptible host ingests a uropathogen and (2) an infectious dose of the uropathogen is transferred from the host’s gastrointestinal tract to his or her urinary tract. As shown above, the first step appears to occur regularly in the community; therefore, the rate-limiting step is expected to be the transfer of the uropathogen to the urinary tract. Given these important distinctions, FUTIs represent a significant shift from the classic foodborne illness paradigm and broadens the implications of antibiotic-resistant *E. coli* in the food supply.

## FUTIs AND ANTIMICROBIAL USE IN POULTRY PRODUCTION

Among the major meat producing species, chickens and turkey appear to be the greatest source of human exposure to antibiotic-resistant ExPEC, and this has important implications regarding antimicrobial use in poultry production. In the US, antimicrobials are administered to poultry as feed and water additives as well as chick and egg (*in ovo*) injections. Even antimicrobials considered critical for human health and treatment of Gram-negative extraintestinal diseases have been used for routine, non-therapeutic purposes in broiler chickens in the US and Canada. For example, day-old chicks and poultry eggs are routinely injected with aminoglycosides (gentamicin) and third-generation cephalosporins (ceftiofur), which has been directly associated with cephalosporin-resistant foodborne infections (Dutil et al., 2010). Given the strong evidence for FUTIs, the critical nature of antimicrobial therapy for treating UTIs, and the clear links between antimicrobial use in FAP and the selection for antimicrobial-resistant *E. coli*, a reevaluation of the antimicrobial classes and antimicrobial applications permitted in this industry is warranted.

There have been some recent, albeit minor, advancements in US agricultural antimicrobial use policy. In 2012, the FDA released new voluntary guidelines discouraging the use of antimicrobials for growth promotion purposes and encouraging the inclusion of veterinary oversight in the application of medically important antimicrobials in food animals (FDA, 2012a). Unfortunately, without any surveillance system in place to ensure compliance, it will be difficult to know if these voluntary guidelines have any positive impact. Likewise, in 2012 the FDA released a final rule restricting extra-label uses of cephalosporins in food-animals, including *in ovo* injection of poultry eggs (FDA, 2012b), but this ruling does not ban the common practice of injecting day-old chicks with cephalosporins. It is important to note that although the US Department of Agriculture (USDA) prohibits the use of antimicrobials in poultry sold under the USDA Organic label, these regulations are only applied starting on day 2 of the animals’ life (USDA, 2012). This is an important loophole that may diminish the distinction between the microbial quality of USDA Organic and conventional products.

## CONCLUSION

As described above, FUTIs represent a major paradigm shift in our understanding of foodborne disease, but require additional research to accurately quantify their contribution to antibiotic-resistant UTIs in general. Of particular value would be studies that integrate contemporaneous, geographically bounded sampling of the foodborne and UTI *E. coli* isolates, advanced molecular techniques to evaluate clonal and temporal relationships, and detailed food consumption surveys from study participants. More basic research could also reveal why ExPEC strains are more prevalent among poultry species as compared to other food-animal species and reveal new opportunities for interventions, such as on-farm ExPEC vaccination programs. Likewise, FUTIs and their potential impact on human health should be considered when evaluating agricultural antibiotic use policies in the US and abroad.

## Conflict of Interest Statement

The authors declare that the research was conducted in the absence of any commercial or financial relationships that could be construed as a potential conflict of interest.
